# Editorial: *Drosophila* as a model to study neurodegenerative diseases

**DOI:** 10.3389/fnins.2023.1275253

**Published:** 2023-08-24

**Authors:** Francesco Liguori, Udai Bhan Pandey, Filomena Anna Digilio

**Affiliations:** ^1^Experimental Neuroscience, IRCCS Fondazione Santa Lucia, Rome, Italy; ^2^Institute for System Analysis and Computer Science (IASI), National Research Council (CNR), Rome, Italy; ^3^Department of Pediatrics, Children's Hospital of Pittsburgh, University of Pittsburgh Medical Center, Pittsburgh, PA, United States; ^4^Research Institute on Terrestrial Ecosystems (IRET), National Research Council (CNR), Naples, Italy

**Keywords:** *Drosophila melanogaster*, neurodegenerative diseases, human disease model, Amyotrophic Lateral Sclerosis, Alzheimer's disease, Parkinson's disease, Spinocerebellar Ataxia Type 3, proteinopathies

Neurodegenerative diseases (NDDs) are incurable and debilitating conditions characterized by progressive loss of selectively vulnerable populations of neurons in brain, spinal cord and peripheral nerves. This causes worsening motor (ataxia), cognitive (dementia) and autonomic dysfunction over time. NDDs impact many families and represent one of the greatest public health burdens worldwide. Most NDDs are due to a combination of genetic and environmental factors. The largest known risk factor is age, so NDDs are becoming more prevalent due to an aging global population, eventually becoming a globally unmanageable problem. There are currently no drugs available to prevent or treat NDDs, and multiple scientific approaches are needed to understand the etiology of these diseases and develop a therapeutic treatment.

An approach toward understanding diseases is to model mechanisms and identify disease-modifying pathways in less complex, but analogous, organisms. The fruit fly *Drosophila melanogaster* is a highly tractable animal for studying NDDs (Ugur et al., 2016). It shares many genes and biological pathways with humans and has a sophisticated nervous system. Beyond a number of experimental advantages, such as short lifespan, numerous progeny and no ethical concerns, *Drosophila* allows answering complicated biological questions through sophisticated genetic experiments, using many mutants and transgenic lines readily available. In fact, many basic and fundamental biological pathways (e.g., notch and circadian rhythm) were discovered in fly models. As a result, *Drosophila* is now being actively employed not only to study *in vivo* the functions of human NDD genes, but also to select and evaluate potential drugs for therapeutic research.

This Research Topic includes 10 original manuscripts which report specific examples or provide a general overview of the value of using *Drosophila* models to contribute to mechanistic and therapeutic studies of NDDs.

Koza et al. used a *Drosophila* model of Parkinson's Disease (PD) to investigate Sexual Dysfunction (SD) which, despite being one of the most common non-motor symptoms of PD, is still poorly understood and studied. PD is a NDD characterized by a series of motor impairments due to a reduction in the number of dopaminergic (DAergic) neurons in the substantia nigra. Leveraging *Drosophila* courtship and climbing behaviors, authors showed that SD precedes motor defects, as well as brain DAergic neurodegeneration and alteration in dopamine metabolism. Interestingly, courtship-related traits could be used as early markers to identify the later onset of PD in the model *Drosophila*.

Rozich et al. presented an optimized protocol, based on the Gal80-DD tool, that allows finely controlled pan-neuronal expression in adult flies of NDD-associated genes, whose manipulation causes developmental lethality in *Drosophila*. Authors tested this method by examining the degenerative phenotypes caused by disruption in the adult brain of the *Vps13D* gene that, in human, causes the ataxia and whose functional inactivation leads to developmental lethality in *Drosophila*.

Prifti et al. used a Spinocerebellar Ataxia Type 3 (SCA3) *Drosophila* model to investigate how specific protein domains of Ataxin3 participate to the toxicity induced by the aberrant form of this protein; Authors reported that mutations in the UbS1 domain enhanced the *in vivo* toxicity of pathogenic Atxn3 through its role in ubiquitin processing and suggest to further explore this domain as a target for therapeutic interventions.

In their Brief Research Report, Borg et al. evaluated the contribution of the *DCTN1* gene in the pathology of Amyotrophic Lateral Sclerosis (ALS), constitutively or tissue-specifically silencing its fly ortholog. Interestingly, authors showed that *Dctn1* and its related paralog (*Dred*) are required for neuronal and muscular function in *Drosophila*.

In their Methods Article, Ayajuddin et al. proposed an easy and inexpensive fluorescence microscopy-based method to quantify neurodegeneration of dopaminergic neurons in a PD *Drosophila* model. The extent of DAergic neurodegeneration was correlated to the fluorescence intensity obtained from tyrosine hydroxylase immunostaining. Present method can also be used to characterize the extent of degeneration of different cell types with little modification and would be of interest to laboratories lacking confocal microscopy.

Varte et al. reviewed how *Drosophila* can contribute to understanding the molecular mechanisms linking mitochondrial dysfunction with Alzheimer's Disease (AD). Authors focused on specific mitochondrial insults caused by amyloid-β and tau in transgenic flies and on the available genetic tools and sensors to study these defects in flies.

Recently, several studies reported the potential benefits of hypertension drugs in AD patients, although their mechanisms of action in the context of AD is unclear. In AD *Drosophila* models, renin-angiotensin system (RAS) inhibitors can suppress neuronal cell death and memory defects even if RAS is not conserved in flies. Ghalayini and Boulianne summarized the studies of renin angiotensin inhibitors in AD and proposed to use *Drosophila* to further elucidate mechanisms underlying RAS system in AD.

Ye et al. summarized novel insights obtained through recent studies examining age and gender as contributing factors to trauma-mediated neurodegeneration in humans and preclinical models, including mammalian and *Drosophila* models. They discussed the central role played by *Drosophila*-based injury models, which offer a unique opportunity not only to study important risk factors associated with NDDs, particularly age and gender, but also to investigate mechanisms underlying head trauma-induced neurodegeneration and to identify therapeutic targets for treatment and recovery.

In their comprehensive review Pan et al. discussed the implications of sphingolipid (SL) metabolism which is affected in a surprisingly broad set of NDDs. These include some lysosomal storage diseases, Friedreich's ataxia, as well as some forms of ALS and PD. Many of these diseases have been modeled in *Drosophila* and are associated with elevated ceramide levels. Authors summarized the elegant research work done in fly models that has advanced our understanding of the nature of defects in SL metabolism, the organelles implicated and potential therapies for these diseases.

Santarelli et al. reported how the use of several *Drosophila* models that overexpress the human mutated genes that cause neuro-proteinopathies (PPs), such as Huntington's Disease (HD), PD, AD, crucially contributed to our understanding of the close relationship between PPs and autophagy. Authors highlighted the importance of these models for studying the function of several risk genes and the benefits of using specific genetic tools in *Drosophila* to generate additional models that will help to better understand the non-autonomous signals exchanged between glia and neuronal cells that could be responsible for some PPs.

## Author contributions

FL: Writing—review and editing. UP: Writing—review and editing. FD: Writing—original draft, Writing—review and editing.
